# The clinical *Pseudomonas fluorescens *MFN1032 strain exerts a cytotoxic effect on epithelial intestinal cells and induces Interleukin-8 via the AP-1 signaling pathway

**DOI:** 10.1186/1471-2180-10-215

**Published:** 2010-08-10

**Authors:** Amar Madi, Omar Lakhdari, Hervé M Blottière, Muriel Guyard-Nicodème, Karine Le Roux, Anne Groboillot, Pascal Svinareff, Joel Doré, Nicole Orange, Marc GJ Feuilloley, Nathalie Connil

**Affiliations:** 1 LMDF-SME, Laboratoire de Microbiologie du Froid-Signaux et Micro-Environnement, UPRES EA 4312, 55 rue Saint Germain, 27000 Evreux, France; 2 BIOGALENYS, 9 Rue de Pacy, 27930 Miserey, France; 3 INRA, UMR 1319 MICALIS, Domaine de Vilvert, F-78352 Jouy-en-Josas, France

## Abstract

**Background:**

*Pseudomonas fluorescens *is present in low number in the intestinal lumen and has been proposed to play a role in Crohn's disease (CD). Indeed, a highly specific antigen, I2, has been detected in CD patients and correlated to the severity of the disease. We aimed to determine whether *P. fluorescens *was able to adhere to human intestinal epithelial cells (IECs), induce cytotoxicity and activate a proinflammatory response.

**Results:**

Behaviour of the clinical strain *P. fluorescens *MFN1032 was compared to that of the psychrotrophic strain *P. fluorescens *MF37 and the opportunistic pathogen *P. aeruginosa *PAO1. Both strains of *P. fluorescens *were found to adhere on Caco-2/TC7 and HT-29 cells. Their cytotoxicity towards these two cell lines determined by LDH release assays was dose-dependent and higher for the clinical strain MFN1032 than for MF37 but lower than *P. aeruginosa *PAO1. The two strains of *P. fluorescens *also induced IL-8 secretion by Caco-2/TC7 and HT-29 cells *via *the AP-1 signaling pathway whereas *P. aeruginosa *PAO1 potentially used the NF-κB pathway.

**Conclusions:**

The present work shows, for the first time, that *P. fluorescens *MFN1032 is able to adhere to IECs, exert cytotoxic effects and induce a proinflammatory reaction. Our results are consistent with a possible contribution of *P. fluorescens *in CD and could explain the presence of specific antibodies against this bacterium in the blood of patients.

## Background

*Pseudomonas aeruginosa *is an opportunistic pathogen frequently emerging from the mucosa-associated intestinal microbiota, which can cause severe septicemia in immuno-compromised hosts. Several interaction mechanisms of *P. aeruginosa *with intestinal epithelial cells (IECs), especially adhesion and penetration, have been studied in detail [1-3]. Conversely, little attention has been given to other species of the same genus, like *Pseudomonas fluorescens*.

*Pseudomonas fluorescens *has long been considered as a psychrotrophic microorganism, unable to grow at temperatures over 32°C, however we have recently shown that some strains isolated from a clinical environment are able to grow at or above 37°C [4]. *P. fluorescens *is a widespread gram-negative bacterium present in a variety of ecological niches such as refrigerated food products, soil, water [5] and in the digestive tract [6]. Interestingly, a highly specific antigen of *P. fluorescens*, designated as I2, was detected in the serum of 54% of the patients suffering from ileal Crohn's disease (CD) [7] and a direct link between the severity of the pathology and the level of circulating I2 antigen has been demonstrated [8]. Surprisingly, the proinflammatory potential of this bacterium or its interaction with the intestinal epithelium has never been investigated.

Several studies have focused on the mucosal immune response to pathogenic bacteria. Human IECs infected with pathogenic bacteria generally produce proinflammatory cytokines, such as interleukin (IL)-8 [9]. The latter has a chemotactic role and can recruit polymorphonuclear cells into the infected site and promote their infiltration of the epithelial layer infected by invasive or noninvasive bacteria [10,11]. IL-8 gene expression is regulated by two major transcriptional factors: nuclear factor kappa B (NF-κB) and activator protein (AP)-1 [12]. NF-κB has a pivotal role in the immune and inflammatory response, but also controls cell survival, proliferation and differentiation [13,14]. Recent works demonstrated that NF-κB signaling is a critical element of the homeostatic immuno-inflammatory function in the gut. Indeed, epithelial NF-κB preserves the integrity of the gut epithelial barrier and coordinates the antimicrobial actions of the innate and adaptive immune systems [15]. Nevertheless, hyperactivation of this transcription factor results in chronic inflammatory bowel diseases [16]. Activation of AP-1 is dependent on mitogen-activated protein kinases (MAPK) that are central in many physiological processes, including regulation of cytokine and stress responses and cytoskeletal reorganization [17,18].

*P. fluorescens *MFN1032 is a clinical strain recently isolated in our laboratory [19]. It displays hemolytic activity toward sheep erythrocytes [20,21], however, its infectious potential on human IECs is still unknown.

In the present study, we investigated adhesion and cytotoxic properties of *P. fluorescens *MFN1032 on Caco-2/TC7 and HT-29 cell lines in comparison to the psychrotrophic strain, *P. fluorescens *MF37 and the well-known opportunist pathogen *P. aeruginosa *PAO1. The proinflammatory potential of *P. fluorescens *MFN1032 was also evaluated by the measurement of IL-8 secretion on both Caco-2/TC7 and HT-29 cells, and analysis of NF-κB and AP-1 activation using the reporter gene strategy.

## Results

### Adhesion to intestinal epithelial cells

**Figure 1:**
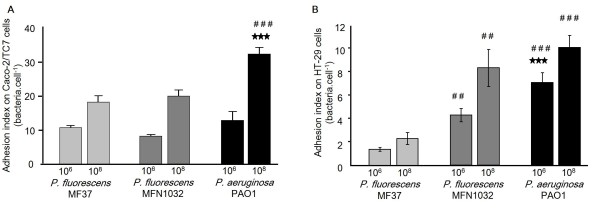
**Adhesion of *P. fluorescens *MF37, *P. fluorescens *MFN1032 and *P. aeruginosa *PAO1 on Caco-2/TC7 (A) and HT-29 (B) cells after 5 h of infection at 10^6 ^or 10^8 ^CFU ml^-1^**. The adhesion index (mean number of bacteria adherent per cell) was calculated by direct microscopic counting of 100 cells. Results were calculated as the mean values (± SEM) of three independent experiments. For each dosis, # # *P *< 0.01 versus MF37, # # # *P *< 0.001 versus MF37, *** *P *< 0.001 versus MFN1032.

The binding index of the clinical strain *P. fluorescens *MFN1032 on Caco-2/TC7 and HT-29 cells was determined after 5 h of incubation and compared to *P. fluorescens *MF37 and *P. aeruginosa *PAO1. The data presented in Figure 1 show that these bacterial strains adhere to both cell lines but the binding index was higher for Caco-2/TC7 (Figure 1A) than for HT-29 (Figure 1B).

*P. aeruginosa *PAO1 showed the highest adhesion potential on Caco-2/TC7 cells compared to *P. fluorescens *MF37 and *P. fluorescens *MFN1032. When the cells were infected with a 10^6 ^CFU or 10^8 ^CFU ml^-1 ^bacterial solution, the mean adhesion index of *P. aeruginosa *PAO1 reached 12.6 ± 2.6 or 32.1 ± 1.9 bacteria cell^-1^, respectively, whereas the adhesion of *P. fluorescens *was quite similar for the two strains with 10.6 ± 0.5 or 18.1 ± 1.9 bacteria cell^-1 ^and 8.2 ± 0.6 or 19.8 ± 2 bacteria cell^-1 ^for MF37 and MFN1032, respectively.

The same experiment using HT-29 cells showed that the binding index of *P. aeruginosa *PAO1 remained the highest (7.1 ± 0.8 or 10.1 ± 1.0 bacteria cell^-1^) but the index *of P. fluorescens *MFN1032 (4.3 ± 0.6 or 8.3 ± 1.6 bacteria cell^-1^) was significantly higher than that of MF37 (1.4 ± 0.2 or 2.3 ± 0.5 bacteria cell^-1^).

### Cytotoxicity assay

**Figure 2:**
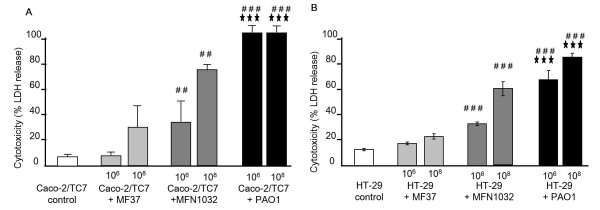
**Cytotoxic effects of *P. fluorescens *MF37, *P. fluorescens *MFN1032 and *P. aeruginosa *PAO1 on Caco-2/TC7 (A) and HT-29 (B) cells**. Cytotoxicity was determined by LDH release assay. Results were calculated as the mean values (± SEM) of three independent experiments. For each dosis, # # *P *< 0.01 versus MF37, # # # *P *< 0.001 versus MF37, *** *P *< 0.001 versus MFN1032.

The cytotoxic effect of *Pseudomonas *strains on Caco-2/TC7 and HT-29 cells was determined by quantification of lactate dehydrogenase (LDH) released in culture medium (Figure 2).

**Figure 3:**
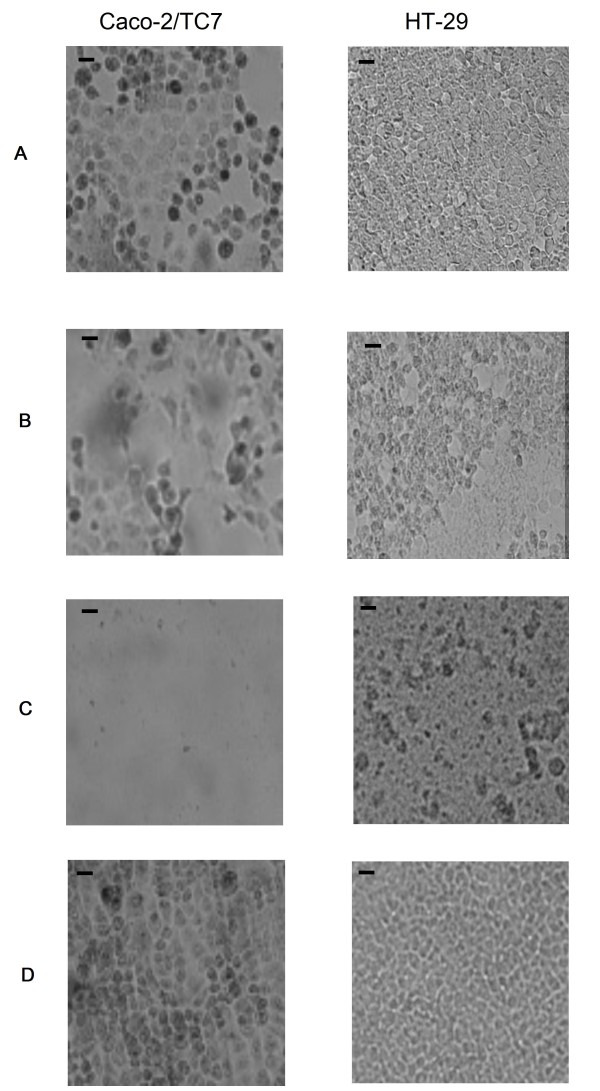
**Effects of *P. fluorescens *MF37 (A), *P. fluorescens *MFN1032 (B) and *P. aeruginosa *PAO1 (C) on the morphological aspect of Caco-2/TC7 and HT-29 monolayers compared to a non-infected monolayer (D)**. The figure only shows the results obtained after 24 h of infection with a concentration of 10^8 ^CFU.ml-^1^. Scale bar = 100 μm.

*P. fluorescens *MF37 exhibited the lowest cytotoxic activity (expressed as % of maximal LDH release) with only 7.8 ± 1.9% (at 10^6 ^CFU ml^-1^) or 30 ± 16.4% (at 10^8 ^CFU ml^-1^) of cell lysis after 24 h of infection on Caco-2/TC7 (Figure 2A) and 17.5 ± 1.1% (at 10^6 ^CFU ml^-1^) or 22 ± 2.0% (at 10^8 ^CFU ml^-1^) of cell lysis for HT-29 cells (Figure 2B). The cytotoxicity of MFN1032 was higher with 34 ± 15.2% or 74.7 ± 4.6% lysis for infection respectively with 10^6 ^or 10^8 ^CFU ml^-1 ^on Caco-2/TC7 and 33.2 ± 1.5 or 60.3 ± 5.5% lysis after infection with 10^6 ^or 10^8 ^CFU ml^-1 ^respectively on HT-29. *P. aeruginosa *PAO1 led to a total lysis of Caco-2/TC7 at the two bacterial concentrations tested and on HT-29, with infection rates of 10^6 ^or 10^8 ^CFU ml^-1^, LDH release was 67.9 ± 7.2% or 85.6 ± 3.4% respectively. At the end of infection, Caco-2/TC7 and HT-29 cells were observed by light microscopy. Figure 3 shows the cell monolayers after infection with MOI of 100 (10^8 ^CFU ml^-1^). When Caco-2/TC7 cells where infected with *P. fluorescens *MF37, a slight cell detachment was detectable while more cells were detaching after infection with MFN1032. Infection with *P. aeruginosa *PAO1 led to a complete disappearance of the organized Caco-2/TC7 and HT-29 monolayers.

### Induction of IL-8 secretion

**Figure 4:**
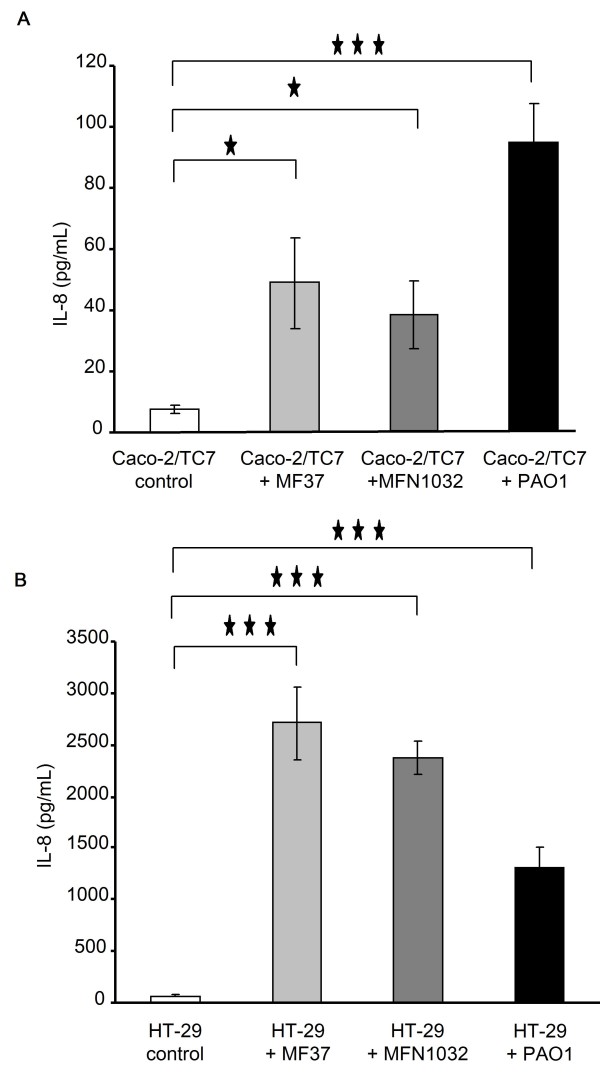
**Induction of IL-8 release by *P. fluorescens *MF37, *P. fluorescens *MFN1032 and *P. aeruginosa *PAO1 in Caco-2/TC7 (A) and HT-29 (B) cells**. IL-8 content was estimated in the cells supernatant after 24 h of infection. * P < 0.05, *** P < 0.001.

The bacterial proinflammatory effect was assessed by measuring IL-8 secretion. Compared to untreated cells, the three *Pseudomonas *strains induced significant stimulation of IL-8 secretion in both Caco-2/TC7 (Figure 4A) and HT-29 cells (Figure 4B). Mean values of IL-8 on HT-29 and Caco-2 in response to *P. fluorescens *MF37 and MFN1032 were similar for these two strains and it is noteworthy that IL-8 secretion was significantly increased in HT-29 compared to Caco-2 cells.

### NF-κB and AP-1 activation in Caco-2 and HT-29 reporter cell lines

**Figure 5:**
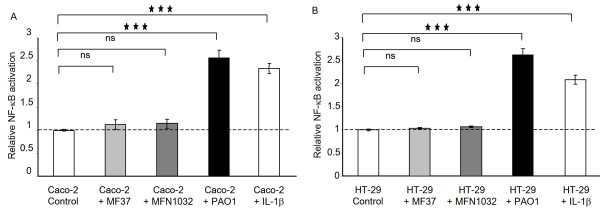
**Effects of *P. fluorescens *MF37, *P. fluorescens *MFN1032 and *P. aeruginosa *PAO1 on Caco-2/κb-seap-7 and HT-29/κb-seap-25 cells expressing an NF-κB/SEAP reporter system**. The relative NF-κB activation corresponding to SEAP activity is expressed in comparison to the activity measured in untreated control cells. IL-1β was used as positive control of NF-κB activation. ns: not significant, *** P < 0.001.

**Figure 6:**
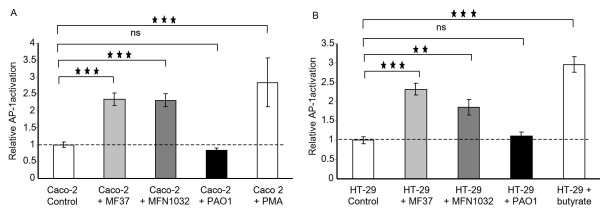
**Effects of *P. fluorescens *MF37, *P. fluorescens *MFN1032 and *P. aeruginosa *PAO1 on Caco-2/ap1-luc-1 and HT-29/ap1-luc-6 cells expressing an AP-1/luciferase reporter system**. The relative AP-1 activation corresponding to luciferase activity is expressed in comparison to the activity measured in untreated control cells. Phorbol-myristate -acetate (PMA) or butyric acid were used as positive controls of AP-1 pathway activation in Caco-2/ap1-luc-1 or HT-29/ap1-luc-6 reporter cells respectively. ns: not significant, ** P < 0.01, *** P < 0.001.

To further explore the immuno-modulatory properties of *P. fluorescens *MFN1032, we tested the effects of this bacterium on NF-κB or AP-1 activation using Caco-2 and HT-29 reporter cell lines. We observed that *P. aeruginosa *PAO1 stimulated NF-κB activity by 2.5-fold over control in both Caco-2/κb-seap-7 and HT-29/κb-seap-25 reporter clones (Figure 5) while it had no effect on the AP-1 pathway (Figure 6). Interestingly, *P. fluorescens *MF37 and MFN1032 had an opposite effect. Indeed, none of these strains induced NF-κB activation (Figure 5) whereas they both activated the AP-1 pathway by 2.2-fold over control in Caco-2/ap1-luc-1 and HT-29/ap1-luc-6 reporter clones (Figure 6).

## Discussion

*P. fluorescens *is present at low level in the human gut and has been linked to Crohn's disease (CD) [7,8], however little is known about the potential interaction of this bacterium with the intestinal mucosa. In the present paper, we aimed at determining its potential to adhere to IEC, to induce cell cytotoxicity and trigger a proinflammatory response. We selected two strains, a classical psychrotrophic strain (MF37) and a recently characterized clinical strain adapted to grow at 37°C (MFN1032). The behaviour of these bacteria was compared to that of the opportunistic pathogen *P. aeruginosa*.

Since adhesion and cytotoxicity to IECs are crucial events in the infection process, the three strains were tested on two epithelial cell lines. Except for adhesion, the two IECs models used in this study gave similar responses to the three strains of *Pseudomonas*. Indeed, a dose dependent adhesion of bacteria to Caco-2/TC7 and HT-29 cells was observed with the greatest effect obtained with the opportunistic pathogen *P. aeruginosa*. It is noteworthy that, compared to the psychrotrophic strain MF37, the clinical strain *P. fluorescens *MFN1032, which is adapted to develop at 37°C displayed statistically significant higher adhesion potential to HT-29 but not to Caco-2/TC7 cells. This observation suggests that the clinical strain may express a greater diversity of adhesion factors than MF37 and could explain, at least in part, the higher cytotoxicity effect of MFN1032.

Although differences exist between surface proteins expressed by Caco-2/TC7 and HT-29 cell lines in comparison to normal human IECs, our results support the hypothesis that *P. fluorescens *should be able to colonize the intestinal mucosa. *Pseudomonad *are rarely searched for and detected as fecal bacteria, and are usually considered as a sub-dominant population [22]. In addition, there is now ample evidence that the circulating bacterial population in the intestinal lumen is very different from the resident microbiota that comes in contact with the apical surface of the enterocytes and is tightly associated to the mucus/glycocalyx layer [23,24]. For an aerobic bacterium such as *P. fluorescens*, the best ecological niche should be at the vicinity of the epithelium, where oxygen concentration is the highest in the intestinal environment [25]. This is supported by the evidence showing that the *P. fluorescens*-specific I2 antigen sequence is systematically detected in ileal mucosa samples [7]. Moreover, in CD patients, there is a positive correlation between blood level of circulating anti-I2 antibodies and the severity of the disease [8] suggesting that the I2-producing bacteria, *i.e. P. fluorescens*, are in close contact with enterocytes and could contribute to CD pathogenesis.

The LDH release assay showed that the cytotoxicity of *P. fluorescens *on Caco-2/TC7 and HT-29 cells is lower than that of *P. aeruginosa*. This limited virulence of the *P. fluorescens *strains seems to be normal for a species that should be a resident in the intestine whereas *P. aeruginosa *is typically an opportunistic pathogen only detected in case of declared infection [26]. This hypothesis is also in agreement with the hierarchy of the cytotoxic activity of the two tested strains of *P. fluorescens*, the clinical strain MFN1032 being more virulent than the environmental and psychrotrophic strain MF37. Bacterial cytotoxicity is a highly complex phenomenon combining the virulence of the prokaryote and the intrinsic sensitivity of the eukaryotic cell. In opposition to the present results, Chapalain *et al *found that the cytotoxic activity on glial cells was higher for *P. fluorescens *MF37 than MFN1032 [4]. These observations are in agreement with the work of Picot *et al *showing that in the case of *P. fluorescens*, the necrotic and apoptotic activities are not simply correlated to the adhesion potential of the strain [27].

In contrast to *P. aeruginosa*, the proinflammatory effect of *P. fluorescens *strains has not been elucidated. In this study, we demonstrated that similarly to *P. aeruginosa*, *P. fluorescens *MFN1032 and MF37 exerted a direct proinflammatory effect on IECs as demonstrated by induction of IL-8 secretion. The homogenous proinflammatory response of IECs induced by the two *P. fluorescens *strains studied suggests a link between the proinflammatory properties and a common pathogenic factor of these strains. IL-8 gene expression is regulated by several signaling pathways including mainly NF-κB and AP-1 transcription factors. Previous studies have shown that *P. aeruginosa *activates NF-κB in mouse monocyte/macrophage cell line [28] and MAPK signaling pathways in lung epithelial cells [29], which in turn leads to the production of proinflammatory cytokines, such as IL-6, IL-8, and TNF-α (tumor necrosis factor alpha). In our study, the two *P. fluorescens *strains failed to activate the NF-κB pathway in contrast to *P. aeruginosa*, however the two strains were able to activate AP-1 signaling, suggesting that the proinflammatory effect of these bacteria in IECs is linked to the activation of MAPK signaling pathways. The MAPK form a group of three pathways, including extracellular signal-regulated protein kinases (ERK1/2) and two stress-activated protein kinases designated p38 and JNK (c-jun N-terminal kinase) [30]. The activation of MAPK has been reported to be involved in response to infection by invasive bacteria, such as *Salmonella enterica *serovar typhimurium or *Listeria monocytogenes*, in IECs [31,32] or in macrophages [33]. Moreover, it has been shown that enteroadherent *Escherichia coli *activate this pathway and both bacterial attachment and secreted proteins might be implicated in cytokine responses [34]. *P. aeruginosa *as well as *P. fluorescens *contain multiple cell surface factors, including flagellin, pili, LPS, type III-mediated toxin secretion and quorum-sensing molecules which could interact with distinct epithelial membrane proteins, such as asialylated glycolipid receptors, toll like receptors (TLRs), or combinations of these proteins [28,35,36]. We have shown that purified flagellin strongly activated NF-κB pathway in HT-29 and to a lower extent in Caco-2, whereas both cell lines poorly responded to LPS (Lakhdari et al, submitted manuscript). In contrast, purified flagellin and LPS do not activated the AP-1 pathway in the two cell lines (data not shown). Thus, we can conclude that *P. fluorescens *activated AP-1 pathway in Caco-2 and HT-29 independently of flagellin and LPS expression. Further investigations will be needed to identify the exact nature and function of *P. fluorescens *compounds responsible for MAPK activation in IECs.

## Conclusions

*P. fluorescens *MFN1032, *P. fluorescens *MF37 and *P. aeruginosa *PAO1 were found to adhere to Caco-2/TC7 and HT-29 cells and the cytotoxicity towards these cell lines was higher for the clinical strain MFN1032 than for MF37. We showed that the two strains of *P. fluorescens *induced IL-8 secretion by Caco-2/TC7 and HT-29 cells *via *the AP-1 signaling pathway whereas *P. aeruginosa *PAO1 potentially used the NF-κB pathway. To our knowledge, this work is the first to demonstrate the interaction and the proinflammatory potential of *P. fluorescens *on IECs.

## Methods

### Cell culture

The human colon adenocarcinoma cell lines Caco-2/TC7 [37] and HT-29 were used between passages 10 and 35. Caco-2/TC7 cells were grown in Dulbecco's modified Eagle Minimal Essential Medium (Sigma) containing 20% foetal calf serum (FCS) supplemented with 2 mM of L-glutamine, 100 U ml^-1 ^each of penicillin and streptomycin and 1% non-essential amino acids at 37°C with 5% CO_2_. HT-29 cells were grown in Dulbecco's modified Eagle Minimal Essential Medium (Sigma) containing 10% FCS supplemented with 2 mM of L-glutamine, 100 U ml^-1 ^each of penicillin and streptomycin at 37°C with 5% CO_2_.

### Bacterial strains and culture conditions

*P. fluorescens *MF37 is a rifampicin-resistant natural mutant of the strain MF0 (Biovar V), originally identified in crude milk [38]. *P. fluorescens *MFN1032, is a clinical biovar I strain collected in a hospital of Haute-Normandie (France) [4]. *P. aeruginosa *PAO1 was obtained from an international collection. Bacteria were grown overnight in ordinary nutrient broth (Merck) at 28°C for the two strains of *P. fluorescens *and at 37°C for *P. aeruginosa *PAO1. For adhesion and cytotoxicity assays, bacteria in stationary phase were harvested by centrifugation (5000 × *g*, 5 min, 20°C) and resuspended in antibiotic-free and serum-free cell culture media at densities of 10^6 ^and 10^8 ^CFU ml^-1^, corresponding to a multiplicity of infection (MOI) of 1 and 100 respectively.

### Adhesion assay

For adhesion assays, Caco-2/TC7 and HT-29 cells were seeded at a concentration of 1 × 10^5 ^cells ml^-1 ^on coverslips coated with 50 *μ*g ml^-1 ^poly-L-lysine and used at 80% confluence as recommended by Li *et al *[39]. Cells were incubated for 5 h with 1 ml of the bacterial suspensions. After incubation, the medium and non-adherent bacteria were removed by washing. Then, the coverslips were fixed with methanol (10 min), stained with Giemsa solution (20 min) and observed using an Axiovert S100TM light microscope (Zeiss). The adhesion index (mean number of bacteria adherent per cell) was determined by direct counting on a minimum of 100 cells following the technique of Darfeuille-Michaud *et al *[40].

### Cytotoxicity assay

Confluent Caco-2/TC7 and HT-29 cells cultivated in 24-well culture plates were infected for 24 h with 1 ml of the bacterial suspensions. At the end of incubation, lactate dehydrogenase (LDH) present in the supernatant was measured in each well using the Cytotox 96^R ^enzymatic assay (Promega). LDH is a stable cytosolic enzyme released by eukaryotic cells and an overall indicator of necrosis. Caco-2/TC7 and HT-29 cells exposed to Triton X100 (0.9%) were used as a control of total release (100% LDH release). The background level (0% LDH release) was determined with serum free culture medium. The percentage of cytotoxicity was calculated following the manufacturer's instructions.

### IL-8 ELISA

IL-8 assays were performed on confluent Caco-2/TC7 and HT-29 cells monolayers grown in 24-well culture plates. After 24 h of infection with the bacterial suspensions (MOI of 100), immunoreactive IL-8 protein levels in cell culture supernatant were quantified using an ELISA Quantikine kit (R&D systems) according to the manufacturer's protocol.

### Construction of stable NF-κB and AP-1 reporter cells

The NF-κB reporter clones Caco-2/κb-seap-7 and HT-29/κb-seap-25 were obtained after a stable transfection of parental cells with the reporter plasmid pNiFty2-SEAP (Invivogen), which contains SEAP (secreted alkaline phosphatase) as reporter gene downstream of five repeats of the NF-κB binding consensus.

The AP-1 reporter clones Caco-2/ap1-luc-1 and HT-29/ap1-luc-6 were obtained after a stable co-transfection of the reporter plasmid pAP-1-luc (Stratagen), which contains luciferase as reporter gene downstream of seven repeats of the AP-1 binding consensus, together with pTK-Hyg (Clonetech) a hygromycine-based selection vector. Transfection of HT-29 was performed by lipofection using TFX-50 (Promega) according to the manufacturer's instructions while Caco-2 cells were transfected using the Amaxa Nucleofector system (Lonza).

### Analysis of NF-κB and AP-1 activation

For each experiment, reporter cells were seeded at 50 000 cells per well, into 96-well plates and pre-incubated 24 hours before adding live bacteria at an MOI of 100.

For NF-κB activation assays, Caco-2/κb-seap-7 and HT-29/κb-seap-25 cells were incubated with live bacteria for 8 hours and IL-1β (10 ng/ml) was used as a positive control. SEAP activity in the supernatant was measured using the Quanti-Blue reagent (Invivogen) using the manufacturer's protocol and quantified as OD at 655 nm.

For AP-1 activation assays, cells were incubated for 12 h with live bacteria. Phorbol-myristate -acetate (PMA, 1 μM) or butyric acid (2 mM) was used as a positive control for Caco-2/ap1-luc-1 or HT-29/ap1-luc-6 reporter cells respectively. Luciferase activity was measured using the ONE-Glo™. Luciferase Assay System (Promega) according to the manufacturer's instructions and quantified as relative luminescence units (RLU).

All measurements were performed using a microplate reader (Infinite 200, Tecan).

### Statistical analysis

Data are expressed as a mean ± standard error (SEM) calculated over three independent experiments performed in triplicate. Analysis of statistical significance were performed by ANOVA with Bonferroni post hoc test (adhesion and cytotoxicity assays) or Student's *t-*test (IL-8 secretion, NF-κB and AP-1 activation assays)

## Authors' contributions

AM carried out most experiments and analyzed most of the data. NC wrote the manuscript, participated in the design of the study and analyzed most of the data. MG carried out the IL-8 ELISA assay. OL carried out the construction of NF-κB reporter cells. KR carried out the construction of AP-1 reporter cells. JD and HB participated in the design of the construction of NF-κB and AP-1 reporter cells and help to draft the manuscript. PS and NO were involved in the design of the study. MF participated in the design of the study and writing of the manuscript, AG performed the statistical analysis. All authors read and approved the final manuscript.
